# Combined test versus logrank/Cox test in 50 randomised trials

**DOI:** 10.1186/s13063-019-3251-5

**Published:** 2019-03-18

**Authors:** Patrick Royston, Babak Choodari-Oskooei, Mahesh K. B. Parmar, Jennifer K. Rogers

**Affiliations:** 1 MRC Clinical Trials Unit at UCL, 90 High Holborn, London, WC1V 6LJ UK; 2 Department of Statistics, University of Oxford, 24-29 St Giles’, Oxford, OX1 3LB UK

**Keywords:** Randomised controlled trials, Time-to-event outcome, Logrank test, Cox test, Hazard ratio, Non-proportional hazards, Combined test, Robustness

## Abstract

**Background:**

The logrank test and the Cox proportional hazards model are routinely applied in the design and analysis of randomised controlled trials (RCTs) with time-to-event outcomes. Usually, sample size and power calculations assume proportional hazards (PH) of the treatment effect, i.e. the hazard ratio is constant over the entire follow-up period. If the PH assumption fails, the power of the logrank/Cox test may be reduced, sometimes severely. It is, therefore, important to understand how serious this can become in real trials, and for a proven, alternative test to be available to increase the robustness of the primary test.

**Methods:**

We performed a systematic search to identify relevant articles in four leading medical journals that publish results of phase 3 clinical trials. Altogether, 50 articles satisfied our inclusion criteria. We digitised published Kaplan–Meier curves and created approximations to the original times to event or censoring at the individual patient level. Using the reconstructed data, we tested for non-PH in all 50 trials. We compared the results from the logrank/Cox test with those from the combined test recently proposed by Royston and Parmar.

**Results:**

The PH assumption was checked and reported only in 28% of the studies. Evidence of non-PH at the 0.10 level was detected in 31% of comparisons. The Cox test of the treatment effect was significant at the 0.05 level in 49% of comparisons, and the combined test in 55%. In four of five trials with discordant results, the interpretation would have changed had the combined test been used. The degree of non-PH and the dominance of the *p* value for the combined test were strongly associated. Graphical investigation suggested that non-PH was mostly due to a treatment effect manifesting in an early follow-up and disappearing later.

**Conclusions:**

The evidence for non-PH is checked (and, hence, identified) in only a small minority of RCTs, but non-PH may be present in a substantial fraction of such trials. In our reanalysis of the reconstructed data from 50 trials, the combined test outperformed the Cox test overall. The combined test is a promising approach to making trial design and analysis more robust.

## Background

The Cox proportional hazards (PH) model is the almost universally used framework for the analysis of time-to-event data in randomised controlled trials (RCTs) with a time-to-event outcome. The primary reason for its popularity is the semi-parametric nature of the model, which assumes only that the hazard functions of two groups remain proportional during the entire follow-up period after randomisation. No parametric form for the baseline hazard is postulated. Departure from PH, if anticipated as a realistic possibility, complicates the design and analysis of such a trial [1]. Generally, the sample size calculation assumes that a logrank or equivalent Cox test will be performed at the analysis stage.

If non-PH is present, the logrank or Cox test may lose power to detect differences between randomised groups. From here on, when we refer to the Cox test, we mean the Cox or logrank test, since they are nearly identical. The magnitude of the loss of power of the Cox test depends on the configuration of non-PH, that is, how the hazard ratio (HR) changes over time [2]. In particular, an early treatment effect, in which the HR differs from 1 relatively early in the follow-up and later approaches or exceeds 1, may severely deplete the power. When estimating the treatment effect in such cases, we argue that because the HR varies over time, non-PH destroys the integrity of a single HR as an adequate and meaningful summary of the treatment effect [3]. We, therefore, disagree with authors (e.g. Schemper et al. [4]) who argue for a time-averaged HR as a useful summary measure in non-PH situations. For example, the HR may even cross between <1 and >1.

Recently, Trinquart et al. investigated the prevalence and implications of non-PH in oncology RCTs reported in five leading journals during the last 6 months of 2014 [5]. They analysed 54 RCTs totalling 33,212 patients. The selected outcome was time to death in 21/54 trials (39%). According to the Grambsch–Therneau test [6], there was evidence of non-PH (*p*<0.1) in 24% of the trials. Further, they empirically compared the treatment effects as measured by the HR and by the difference and ratio of restricted mean survival times (RMSTs) at the most extreme event time. They concluded in favour of routine reporting of RMST measures, whether or not the PH assumption held.

Here, we aim to confirm Trinquart et al.’s findings [5] on the occurrence of non-PH in a further selection of real trials. We also assess the performance of the recently proposed combined test of the treatment effect [2] compared with the Cox test. The combined test may be used to design the power or sample size and analyse the treatment effect in trials in which non-PH could occur [7].

Briefly stated, the combined test aims to exploit the Cox test (which is optimal under PH) and a permutation test of the maximal difference in RMST between treatment groups across several predefined time points. The RMST at a given time does not require any distributional assumption, such as PH. The combined test comprises the minimum of the Cox and permutation test *p* values, corrected for their correlation. The test may detect, for example, a significant early difference between the survival curves even when there is non-PH and the HR is not far from 1.

Our predefined time points for RMST evaluation are the ten equally spaced times between the 30th and the 100th centiles of the uncensored event times. We chose the 30th centile because we felt that very early differences in survival were unlikely to be reliable or of clinical interest. The 100th centile was chosen because it provides good power with divergent survival curves, e.g. under PH. Ten values give reasonable coverage of the curve of *p* values for tests of RMST differences. For further insight into the RMST and RMST difference, we refer the reader to Fig. 2 of Dehbi et al. [8] and Fig. 3 of Royston and Parmar [3].

The structure of the article is as follows. In ‘Methods’, we outline the strategy for selecting eligible trials, the methodology used to extract data from published Kaplan–Meier curves and to reconstruct individual-level time-to-event data, and the Royston–Parmar combined test. In ‘Results’, we report the outcome of the journal search, the assessment of non-PH and the application of the Cox test and the combined test to the reconstructed data. We end with a Discussion and our Conclusions.

## Methods

### Data source and selection procedure

We identified eligible phase III RCTs reported in four leading general medical journals in 2013: the New England Journal of Medicine (NEJM), the British Medical Journal (BMJ), the Lancet and the Journal of the American Medical Association (JAMA). The trials in our study differed from those in Trinquart et al. [5]’s study by publication date and by the range of disease areas considered. We did not limit eligibility to oncology trials. The selection procedure was as follows.

We included superiority trials where the primary outcome was subjected to a time-to-event analysis. Our search string identified 586 potential articles. Two authors (JKR and BCO) independently reviewed the abstract, full text and (in some circumstances) the articles’ supporting material. Consensus was reached by discussion. We excluded analyses using pooled data from two or more trials and reports of secondary, subgroup or follow-up analyses. Of the 586 articles examined, 50 satisfied the following inclusion criteria: main study publication, phase III superiority trial, primary outcome was time to event, and the primary test of the null hypothesis was Cox or logrank. Five multi-arm trials were included, one 4-arm trial of macular degeneration, and four 3-arm trials of HIV, cancer, MRSA and cardiovascular disease.

### Data extraction and reconstruction

We extracted information on type of disease, sample size, median follow-up time, primary endpoint, sample size and number of events. We ascertained whether a test of non-PH had been carried out, and if so, we recorded the type of test. We determined whether the PH assumption was violated, the nature of the violation and which (if any) methods for handling non-PH were considered. Finally, we noted whether a logrank or Cox test had been performed.

As in the procedure followed by Trinquart et al. [5], we reconstructed individual participant data (IPD) for all patients in each treatment group from published Kaplan–Meier curves. We used the DigitizeIt graphical digitisation package (https://www.digitizeit.de/) to read off the time and survival probability coordinates from the Kaplan–Meier curves. Where possible, we extracted the numbers of patients at risk and the total number of events. We estimated individual times to event or censoring by using the community-contributed Stata program ipdfc [9]. The method is based on an algorithm in R described by Guyot et al. [10].

Kaplan–Meier curves were digitised and IPD were reconstructed by an independent person under the supervision of BCO. We made informal visual checks of reconstructed Kaplan–Meier curves compared with those in the original publication, with satisfactory results. In an informal assessment, we found good agreement between the published estimates of the HR and its 95% confidence interval and those from the data produced by ipdfc.

### Combined test of the treatment effect

Under PH, the Cox test has optimal power. The motivation for the combined test is to capitalise on the strength of the Cox test when PH is (nearly) satisfied and to provide insurance (extra power) for cases in which it is not. Under some patterns of non-PH, the power of the Cox test is reduced, even drastically. We aimed to boost the power under such circumstances by combining the Cox test with a suitable additional test—hence the name ‘combined test’.

More generally, the standard null hypothesis in trial design is *H*_0_: HR=1 against the alternative *H*_1_: HR=*δ*. Usually *δ*<1, meaning a reduction (for example) in the mortality rate due to the research treatment. It may happen that *H*_0_ is not rejected at some predefined level *α* but that there are substantial, clinically relevant differences between the two survival curves. In view of the enormous costs and effort involved in mounting a phase III RCT, we would wish to avoid the conclusion that the treatment effect was non-significant and therefore, that the trial was negative solely because *p*>*α* on a test of the primary outcome that does not cover a wide enough range of relevant alternative hypotheses, that is of patterns of non-PH.

The challenge due to the limitations of the PH restriction has been recognised in the literature. Although in no way new, one approach, the RMST, has gained ground as a summary measure of a survival function and for comparing two survival curves. See, for example, A’Hern [11] for an argument for its use in oncology trials. RMST is the mean of a time-to-event distribution truncated at a specific time point, sometimes denoted by *t*^∗^. RMST has a clear interpretation. For example, with *t*^∗^=3 yr, an RMST of 2.5 yr for a group of patients implies that when followed up for 3 yr, on average patients survive 2.5 yr.

The RMST is easily estimated as the area under the corresponding survival curve (e.g. a Kaplan–Meier curve) up to *t*^∗^. Unlike the HR, which is dependent on the model, the treatment effect may be quantified by the difference in RMST values at *t*^∗^, which requires no modelling assumption. Once *t*^∗^ has been selected, significance testing of the difference in RMST between groups is straightforward.

A potential weakness with such a use of RMST is the choice of *t*^∗^. In a clinical trial paradigm, for a single test of RMST difference to be regarded as valid, *t*^∗^ must be prespecified at the design stage. Under PH, a choice of *t*^∗^ relatively late during the follow-up may confer power comparable to that of the Cox test [12], but better choices of *t*^∗^ may considerably increase the power under various patterns of non-PH. To accommodate this feature, Royston and Parmar [2] suggested testing the RMST difference at several prespecified values of *t*^∗^ during the follow-up, taking the smallest *p* value as the basis of a test. They provided a method based on a permutation test to correct the resulting *p* value, which is obviously too small. Lastly they took the smaller of the corrected *p* value and that from the Cox test to give a putative *p* value, again requiring correction for multiple testing. The final result was *p*(CT), the *p* value for the combined test, which has approximately the correct distribution under the global null hypothesis of equal survival curves, *H*_0_: *S*_0_(*t*)=*S*_1_(*t*). Details of the approach are given in [2]. An implementation of the combined test in Stata is described by Royston [13]. Power and sample size calculations for the combined test have been implemented for Stata users by Royston [7].

## Results

### Characteristics of eligible articles

**Fig. 1:**
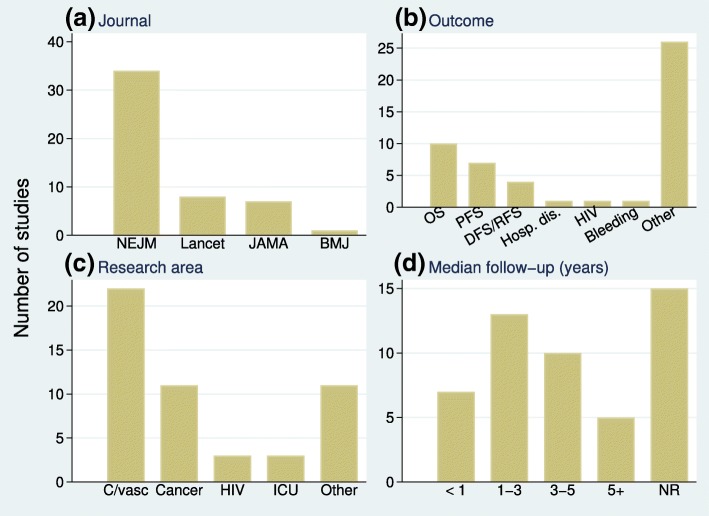
Frequencies of articles. By **a** journal, **b** type of time-to-event outcome, **c** research area and **d** median follow-up time. BMJ *British Medical Journal*, C/vasc cardiovascular disease, DFS/RFS disease-free or recurrence-free survival, HIV human immunodeficiency virus, ICU intensive care unit, JAMA *Journal of the American Medical Association*, NEJM *New England Journal of Medicine*, NR not reported, OS overall survival, PFS progression-free survival

The selected articles cover a wide spectrum of disease research. Most studies (68*%*) came from the NEJM, with only one eligible article from the BMJ (Fig. 1a). Figure 1b describes the types of primary outcome. Composite outcomes, commonly used for cardiovascular disease, were the most frequent. Research areas were grouped into five broad categories: cardiovascular, cancer, HIV, intensive care unit and other. Cardiovascular disease (44%) and cancer (22%) were the two most frequent categories (Fig. 1c). Figure 1d shows the distribution of median follow-up times in the eligible articles. In 28% of articles, the median follow-up time was not reported.

### Reported assessment of the PH assumption

In 36/50 studies (72%), the PH assumption was not assessed (or at least, not reported). For the 14 articles in which it was assessed, the most frequent method (50%) was testing the interaction between the (log of) event time and treatment (Table 1). In five articles, evidence for non-PH was assessed through scaled Schoenfeld residuals from a Cox model. In four of the five articles, graphical methods appear to have been used, with no formal method of assessment, and in one, a formal test (Grambsch–Therneau) was applied. In the Grambsch–Therneau test [6], scaled Schoenfeld residuals are correlated with uncensored event times.

**Table 1:** Methods used to assess the PH assumption

Method of checking non-PH	No. of studies
Treatment × time interaction (likelihood ratio test)	7
Schoenfeld residuals (graphical assessment)	4
Schoenfeld residuals (Grambsch–Therneau test)	1
Method not reported	2
No check done	36
Total	50

*PH* proportional hazards

Among the 14 (28%) articles in which an assessment was carried out, violations of the PH assumption were reported in 2/14 (14%) studies. In one of the two studies, a post hoc non-PH analysis was performed. In the other study, contingent on finding non-PH, as the primary analysis, the follow-up was truncated after a prespecified cut-off time before applying the Cox PH model. In seven articles, the outcome of the PH assessment was not reported. In no study was there evidence of a prespecified plan to cope with a potential non-PH treatment effect.

### Reanalysis of the 50 trials

**Fig. 2:**
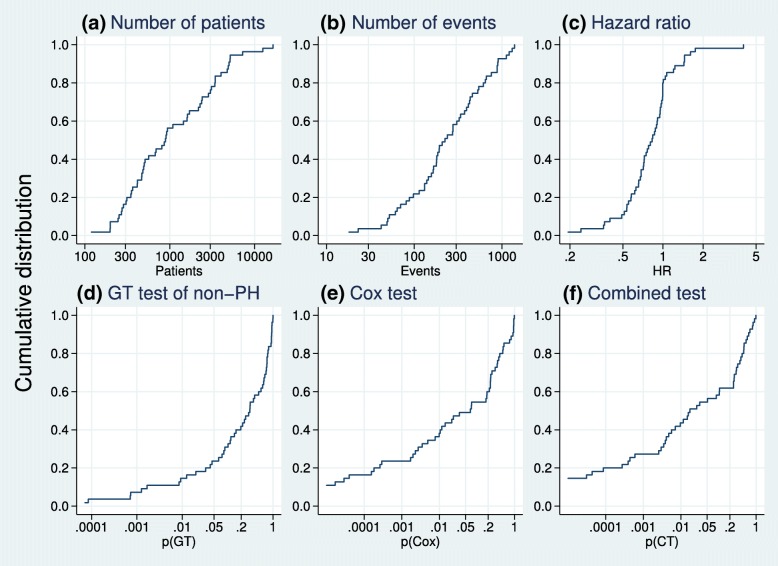
Cumulative distributions of basic data and results from reanalysis of IPD of 55 pairwise treatment comparisons from the 50 trials. Values on the horizontal axis in (**d**), (**e**) and (**f**) are *p* values from the respective tests and are presented on logarithmic scales. IPD individual participant data, PH proportional hazards

We performed Cox and combined tests and the Grambsch–Therneau test of non-PH on data for 55 pairwise comparisons of a research arm with control in the 50 trials. The numbers of patients and events, and the results for each comparison, derived from the reconstructed IPD, are summarised as cumulative distributions in Fig. 2.

The trial sizes ranged between 119 and 16,492, and the numbers of events between 18 and 1390. The estimated HR was <1 in 44/55 comparisons. The Cox test of the treatment effect was significant at the 0.05 level in 27 comparisons (49%). There is evidence of non-PH at the 0.10 level (0.05) in 17 (13) comparisons. The proportion of trials with non-PH significant at the 0.10 level (31*%*) is slightly higher than the 13/54 (24%) reported by Trinquart et al. [5].

### Comparison of Cox and combined tests

Table 2 compares the performance of the Cox and combined tests at a conventional 0.05 significance level. Of the five discordant trials reported in Table 2, the Cox test has *p*<0.05 in one comparison (trial 6) whereas the combined test has *p*<0.05 in four comparisons (from four 2-arm trials). The four trials in which the combined test dominates were on cardiovascular medicine (two trials), dermatology and cancer. The fifth trial in which the Cox test is better was on gastrointestinal disease.

**Table 2:** Comparative performance of the Cox and combined tests

Combined test	Cox test with *p*<0.05		
with *p*<0.05	No	Yes	Total
No	24	1	25
Yes	4	26	30
Total	28	27	55

**Fig. 3:**
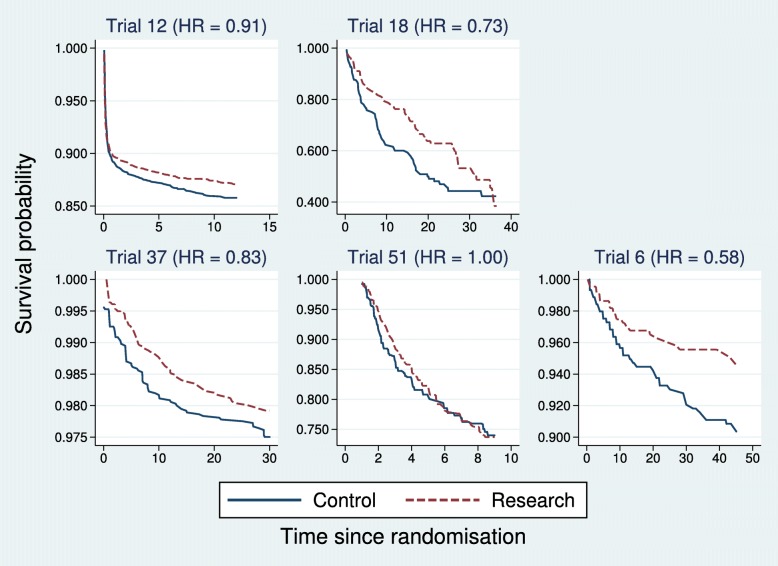
Kaplan–Meier curves for five trials with discordant *p* values from the Cox and combined tests of the treatment effect. For legibility, the vertical scales have been expanded to accommodate the observed ranges of the survival functions. HR hazard ratio

A detailed comparison of survival curves and other summary information in the five discordant trials is shown in Fig. 3 and Table 3. In all except trial 18, the event rate is low (see *S*_min_ in Table 3). There is evidence of non-PH in all four trials in which the combined test is superior (Table 2), but not in trial 6, where the Cox test is superior. Note, however, that the combined test is borderline significant (*p*(CT)=0.051) in trial 6, so the possible power advantage of the Cox model here is small.

**Table 3:** Statistics and tests for the five discordant trials

Trial	Patients	Events	*S*_min_	HR	Test *p* values		
					GT	Cox	Combined
12	4752	645	0.864	0.911	0.001	0.235	<0.001
18	274	131	0.412	0.725	0.045	0.067	0.026
37	7244	164	0.977	0.828	0.033	0.227	0.012
51	3105	756	0.738	0.995	<0.001	0.946	0.007
6	889	64	0.922	0.579	0.766	0.034	0.051

*S*_min_ is the smallest observed value of the Kaplan–Meier survival curve for all patients *GT* Grambsch–Therneau test, *HR* hazard ratio

**Fig. 4:**
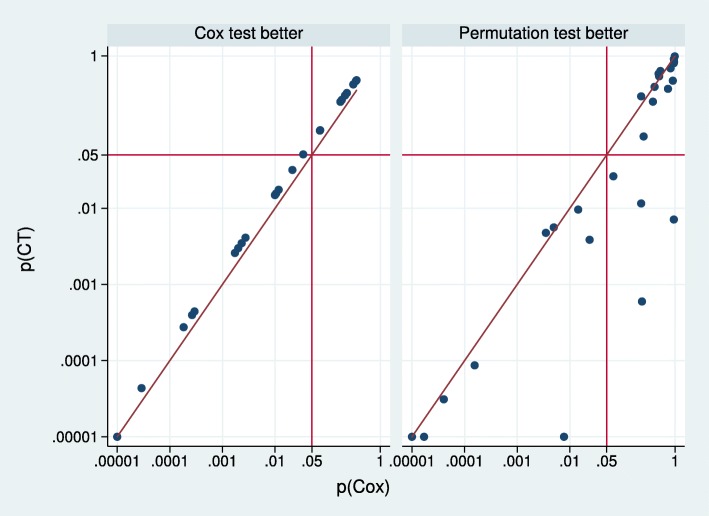
Comparison between combined and Cox test *p* values in 55 randomised treatment-effect comparisons. Values <0.00001 are truncated to 0.00001. The diagonal line is the line of identity. The vertical and horizontal scales are logarithmic. CT combined test

Figure 4 compares the combined and Cox tests, split according to whether the Cox or permutation test has the smaller *p* value. The *p* value for the combined test is a transformation of the smaller of *p*(Cox) and *p*(perm), the *p* value from the permutation test. Note that *p*(CT) is an incomplete beta-function transformation of *p*(min)= min(*p*(Cox),*p*(perm)) [2]. In effect, *p*(CT)=*f*×*p*(min) where the factor *f* lies in (1,1.5), with *f*→1 as *p*(min)→1 and *f*→1.5 as *p*(min)→0.

When *p*(Cox)<*p*(perm) (left panel), *p*(CT)=*f*×*p*(Cox). Since *f* lies in (1,1.5), *p*(CT) exceeds *p*(Cox), but never by more than 50%. In this case, *p*(CT) is heavily constrained by *p*(Cox). If the Cox model is the correct choice (i.e. the PH assumption is true), use of the combined test instead of the Cox test inflates the *p* value by <50*%* and reduces the power. Royston and Parmar [2] illustrated that the power of the combined test under PH may be restored at the design stage by a modest increase in the sample size, typically around 7 to 8%.

The right panel of Fig. 4 compares *p*(CT) with *p*(Cox) when *p*(perm)<*p*(Cox). In most (19) of the 31 comparisons in which *p*(perm)<*p*(Cox), the combined test exhibits an advantage over the Cox test. In this case, *p*(CT) is *not* heavily constrained by *p*(Cox). *p*(CT) may be much smaller than *p*(Cox) and the combined test presumably then is the more powerful.

Table 4 shows the distribution of the ratio *r*=*p*(CT)/*p*(Cox) according to whether there is evidence of non-PH, the criterion *p*(GT)<0.1 used by Trinquart et al. [5]. When *r*<1, the combined test is more significant than the Cox test, and vice versa. The two results with *r*>1 and *p*(GT)≤0.5 are from trial 30 (comparison 2) and trial 49.

**Table 4:** Distribution of *p* value ratio *r* across 55 comparisons in 50 randomised trials

Ratio (*r*)	Evidence of non-PH		Total
*r*=*p*(CT)/*p*(Cox)	No (*%*)	Yes (*%*)	
*r*≤0.5	2 (15)	11 (85)	13 (100)
0.5<*r*≤1.0	7 (64)	4 (36)	11 (100)
*r*>1.0	29 (94)	2 (6)	31 (100)
Total	38 (69)	17 (31)	55 (100)

Based on evidence of non-PH of the treatment effect, defined by *p*(GT)<0.1. *r*<1 favours the combined test

*CT* combined test,

*GT* Grambsch–Therneau test,

*PH* proportional hazards

When there is evidence of non-PH (17/55 comparisons, 31%), the combined test is more significant in 15/17 (88%) of them. In contrast, when *p*(GT)≥0.1 (38/55 comparisons, 69%), the Cox test is more significant in 29/38 (76%) of them. Thus, the relative performance of the tests is associated with the strength of evidence for non-PH, at least according to *p* value criteria. As already mentioned, in such an analysis, the relative performance of the Cox test is mathematically limited, since *r* is bounded above by 1.5 but below by 0.

### Investigation of time-dependent HRs

**Fig. 5:**
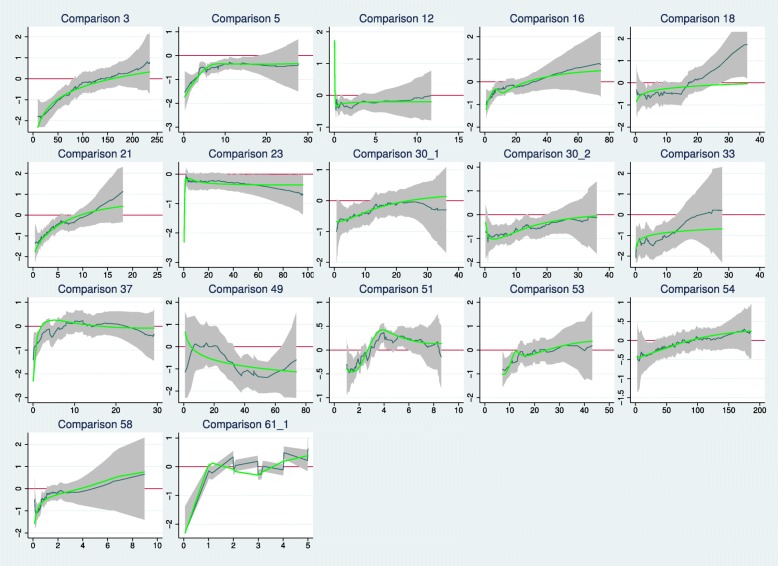
Time-dependent log hazard ratios (green curves) in the 17 comparisons with some evidence of non-PH. Horizontal lines represent logHR=0. See text for further details. HR hazard ratio, PH proportional hazards

It appears that the combined test is more powerful when there is evidence of non-PH. Here we investigate the patterns of HR (*t*), the time-dependent HR in the 17 trials with *p*(GT)<0.1. Figure 5 shows estimates of HR (*t*), based on two methods of analysis. The thin black lines are smoothed scaled Schoenfeld residuals from a Cox model on the treatment covariate, together with pointwise 95% confidence intervals (shaded). The original points (not shown) estimate lnHR(*t*) at the event times. The thicker green lines are estimates of lnHR(*t*) derived from Royston–Parmar survival models [14–16] with the degrees of freedom chosen to minimise the Bayes information criterion. Equal candidate degrees of freedom in the range 1 to 4 for the baseline and time-dependent treatment effect were used. The horizontal red lines denote lnHR(*t*)=0, i.e. HR(*t*)=1.

The predominant visual impression is that the plotted lines tend to increase from a negative region (denoting a lower hazard) to a region with lnHR(*t*)≥0 (zero treatment effect or a higher hazard in the control arm). The one exception is comparison 49, which seems to show a late treatment effect. In confirmation, a time-dependent Cox model with two periods (*t*<40 and *t*≥40 months) gives estimated HRs of 0.77 (standard error 0.28) and 0.31 (standard error 0.21), respectively, consistent with a late effect. However, with a total of only 50 events, this trial is small.

In general terms, the findings suggest that departures from PH, at least in the sample of trials we examined, often exhibit as an early treatment effect. Such trials are precisely the ones for which we expect the combined test to be more powerful than the Cox test [2]. The interpretation is borne out by the analysis of *p* values presented in ‘Comparison of Cox and combined tests’ (Table 2). Furthermore, in comparison 49, *p*(Cox)=0.021 and *p*(CT)=0.032, consistent with our expectation that the Cox test is more powerful than the combined test under a late treatment effect [2]. Qualitatively, however, the results of the two tests lead to the same conclusion.

**Fig. 6:**
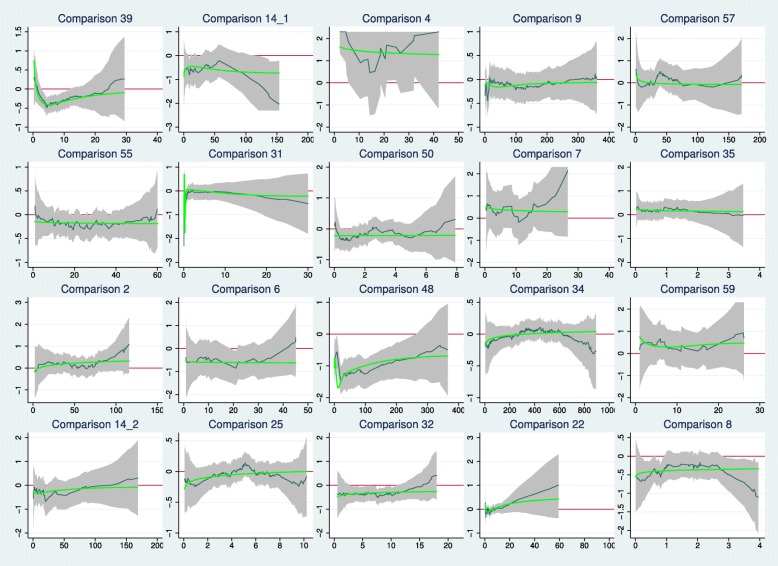
Time-dependent log hazard ratios in the 20 comparisons with *p* values from the Grambsch–Therneau test nearest to 1.0

Figure 6 presents plots similar in construction to Fig. 5, but here for the 20/38 comparisons with *p*(GT)>0.1 and closest to 1.0. The values of *p*(GT) range in row order from 0.998 (comparison 39) to 0.620 (comparison 8). According to the test, they exhibit the least evidence of departure from PH in the sample of 55 comparisons.

Subjectively, we see little evidence of an early (or late) treatment effect. Sometimes, a quadratic type of shape is seen. Because the Grambsch–Therneau test is based on a linear correlation between scaled Schoenfeld residuals and event times, by design it is sensitive to monotonic relationships between HR (*t*) and *t* and has low power against quadratic alternatives.

## Discussion

Our primary aim in this report was to compare the performance of the combined test with that of the Cox test, which is essentially the same as the logrank test. To do this, we sought a realistic database of RCTs in the medical specialities in which many such trials are performed. Further, we needed IPD. We satisfied the first requirement through a systematic review that selected relevant articles published in four key journals in 2013. The second we achieved by applying a relatively recent technology consisting of digitising Kaplan–Meier curves followed by a statistical algorithm devised by Guyot et al. [10] to generate realistic IPD values. We were able to confirm the finding of Trinquart et al. [5] of unrecognised but potentially important non-PH among survival curves in such trials.

Overall, our reanalysis of the reconstructed IPD showed that the combined test indicated a treatment effect significant at the 0.05 level more often than the Cox test. In earlier simulation work [2], the power of the combined test was found to be greater when an early treatment effect was present. This observation was confirmed in the present study. Graphical analysis of scaled Schoenfeld residuals (Figs. 5 and 6), a tool used to investigate the relation of the HR with follow-up time, suggested that an early treatment effect was often present, even when non-PH was not formally significant according to the Grambsch–Therneau test.

In defining the combined test, we replaced the standard null hypothesis of HR =1 with the more general formulation *S*_0_(*t*)=*S*_1_(*t*), that is, with identical survival functions in the control and research arms. A plethora of tests of the equality of two survival curves has appeared in the literature. It is beyond the scope of the present article to make comparisons with other such tests, which remains a topic for further research.

## Conclusions

Using similar technology, we confirmed the finding of Trinquart and colleagues that the evidence for non-PH is checked (and hence, identified) in only a small minority of RCTs, but that non-PH may be present in a substantial fraction. In our reanalysis of the IPD reconstructed from Kaplan–Meier estimates of survival functions in 50 trials, the combined test outperformed the Cox test overall. The combined test is a promising approach to making trial design and analysis more robust, warranting further investigation of its properties, strengths and weaknesses. For example, the performance of the combined test across a wider range of non-PH cases needs to be explored.

Finally, our study and that of Trinquart highlight how important it is for triallists who are designing the sample size for a time-to-event study to consider carefully whether the PH assumption is likely to hold in their research context. The same applies whether, for example, existing literature or clinical expert information is utilised. If the PH assumption turns out to be correct, use of a combined test will lead to a small loss in power. However, if the PH assumption is wrong, use of the Cox or logrank statistical test may result in a substantial loss of power and a misleading time-averaged HR.

## Abbreviations

BMJ: *British Medical Journal*
GT: Grambsch–Therneau test
HIV: Human immunodeficiency virus
HR: Hazard ratio
ICU: Intensive care unit
IPD: Individual participant data
JAMA: *Journal of the American Medical Association*
MRC: Medical research council
MRSA: Multiply resistant *staphylococcus aureus*
NEJM: *New England Journal of Medicine*
PH: Proportional hazards
RCT: Randomised controlled trial
RMST: Restricted mean survival time
UCL: University College London
